# Efficacy of a telephone outcall program to reduce caregiver burden among caregivers of cancer patients [PROTECT]: a randomised controlled trial

**DOI:** 10.1186/s12885-017-3961-6

**Published:** 2018-01-08

**Authors:** Leila Heckel, Kate M. Fennell, John Reynolds, Anna Boltong, Mari Botti, Richard H. Osborne, Cathrine Mihalopoulos, Jacquie Chirgwin, Melinda Williams, Cadeyrn J. Gaskin, David M. Ashley, Patricia M. Livingston

**Affiliations:** 1 Deakin University, Faculty of Health, School of Nursing and Midwifery, Geelong, VIC 3220 Australia; 2 Deakin University, Faculty of Health, School of Health and Social Development, Geelong, VIC 3220 Australia; 3 Cancer Council SA, 202 Greenhill Road, East wood, South Australia 5063 Australia; 4 Flinders Centre for Innovation in Cancer, School of Medicine, Flinders University, Sturt Road, Bedford Park, South Australia 5042 Australia; 5 Sansom Institute for Health Research, University of South Australia, City East Campus, North Terrace, Adelaide, South Australia Australia; 6 Monash University, Faculty of Medicine, Nursing and Health Sciences, Clayton, VIC 3168 Australia; 7 Cancer Council Victoria, 615 St Kilda Road, Melbourne, VIC 3004 Australia; 8 The University of Melbourne, Grattan Street, Parkville, VIC 3052 Australia; 9 Epworth HealthCare, Richmond, VIC 3121 Australia; 10 Eastern Health, Department of Oncology, Box Hill, VIC 3128 Australia; 11 University of Newcastle, Faculty of Health and Medicine, Callaghan, NSW 2308 Australia; 12 Barwon South Western Regional Integrated Cancer Service, Geelong, VIC 3220 Australia; 13 Deakin University, Faculty of Health, Biostatistics Unit, Geelong, VIC 3220 Australia; 14 The Andrew Love Cancer Centre, Geelong, VIC 3220 Australia

**Keywords:** Telephone intervention, Caregivers, Cancer, RCT, Caregiver burden, Unmet needs, Depression, Health literacy, Helpline, Support

## Abstract

**Background:**

Informal caregivers provide extended support to people with cancer but they receive little support from the health care system to assist them in their caring role. The aim of this single-blind, multi-centre, randomised controlled trial was to test the efficacy of a telephone outcall program to reduce caregiver burden and unmet needs, and improve psychological well-being among cancer caregivers, as well as evaluating the potential impact on patient outcomes.

**Methods:**

Cancer patient/caregiver dyads (*N* = 216) were randomised to a telephone outcall program (*n* = 108) or attention control group (*n* = 108). The primary outcome was self-reported caregiver burden. Secondary endpoints included depressive symptoms, unmet needs, self-esteem, self-empowerment, and health literacy. Data were collected at baseline and at both 1 and 6 months post-intervention. An intention to treat analysis was performed.

**Results:**

The intervention had no effect on the primary outcome (caregiver burden), but reduced the number of caregiver unmet needs (intervention group baseline, mean = 2.66, 95% confidence interval (CI) [1.91–3.54]; intervention group 1 month post intervention, mean = 0.85, 95%CI [0.42–1.44]; control group baseline, mean = 1.30 95%CI [0.80–1.94], control group 1 month post intervention, mean = 1.02 95%CI [0.52–1.69]; *p* = 0.023). For caregivers at risk for depression, the intervention had a significant effect on caregivers’ confidence in having sufficient information to manage their health (*p* = 0.040). No effects were found for patients’ depressive symptoms, unmet needs, self-empowerment, and other health literacy domains.

**Conclusions:**

While caregiver burden was not reduced, the outcall program was effective in reducing unmet needs in caregivers. Provision of cancer information and support via a telephone service may represent a feasible approach to reducing unmet needs among cancer caregiver populations.

**Trial registration:**

ACTRN12613000731796; prospectively registered on 02/07/2013.

**Electronic supplementary material:**

The online version of this article (10.1186/s12885-017-3961-6) contains supplementary material, which is available to authorized users.

## Background

With the rise of cancer globally [1], the number of informal caregivers who provide uncompensated care to cancer patients is increasing proportionally [2]. In addition to their existing obligations, caregivers are often confronted with role transitions and new responsibilities of managing the needs of the person diagnosed with cancer [3, 4]. There is increasing recognition that informal caregivers of people with cancer need information and support from the health care system [5, 6]. Failure to address these needs can impact substantially on caregivers’ health causing considerable burden, anxiety, and depression [5, 7–10].

Since the Australian Clinical Practice Guidelines for Psychosocial Care of Adults with Cancer were introduced [11], no recommendations have been made for systematically supporting cancer caregivers. Although psychosocial interventions have been developed to support caregivers (e.g. [12–14]), a meta-analysis of 29 randomised controlled trials comprising psychoeducation, skills training, or therapeutic counselling interventions found only small to moderate effects but significantly reduced caregiver burden and improved aspects of quality of life [15]. Since then there have been calls for other tailored interventions to be designed and tested, particularly for those caring for patients in the early stages of the cancer trajectory [16, 17].

To address the gap in the literature, we linked a high quality and credible telephone-based information and support service (13 11 20) with caregivers who were caring for people recently diagnosed with cancer, in the early stages of treatment. Cancer Council’s 13 11 20 Information and Support is a free telephone service providing tailored support to people affected by cancer across Australia. At present, individuals need to initiate contact with the 13 11 20 service. The aim of this study was to evaluate whether a new model of service delivery (i.e. a telephone outcall program), provided by the established 13 11 20 service, reduced caregiver burden and unmet needs, and improved psychological health among caregivers of people newly diagnosed with cancer.

## Methods

### Research design

This study was a single blind, multi-centre, randomised controlled trial. A detailed description of the study methods is available in the published protocol [18]. Briefly, patient/caregiver dyads were recruited at one private and three public health services in Melbourne and Adelaide, Australia, between August 2013 and December 2014. Eligible dyads were approached by trained research personnel at oncology outpatient units during treatment cycles 2–5 of adjuvant chemotherapy and/or fractions 2–10 of radiotherapy. A brief introduction to the trial was provided and interested dyads were given an information package to take home and followed up to confirm participation. Consenting dyads completed surveys at baseline and at 1 and 6 months post-intervention.

### Participants

Eligible patients were identified by nurses through hospital patient management systems and included those with a primary cancer diagnosis (any cancer type, stages I-III) who received treatment with curative intent. Minimum age of both patients and caregivers was 18 years and dyads had to be able to read and understand English language and present with no cognitive impairment to participate in the study. Caregivers were nominated by the patient as the person most involved in providing support throughout the illness trajectory.

### Cancer council 13 11 20 information and support service

Cancer Council Australia are the largest non-government provider of cancer support services (http://www.cancer.org.au/). Their signature service is the 13 11 20 telephone information and support service, run by specialist oncology nurses with extensive clinical and counselling experience, which they use to educate, support and link callers to other internal and external services, depending on their needs.

### Randomisation and group allocation

A computer-generated randomisation table stratified by health service was produced by the trial statistician and made accessible to the study co-ordinator who conducted the randomization. Dyads were randomised to the intervention or the attention control group and participants were blinded after group allocation. In the intervention group, caregivers received three calls from a 13 11 20 nurse with the first outcall at the start of the program (5–10 days post-randomisation), the second outcall 1 month later and the last outcall 3 months following the second call. During each outcall, the nurse measured caregivers’ distress using the Distress Thermometer [19] and offered referral to appropriate services to those with elevated scores (distress ≥4 and impact ≥3). The nurse then raised six topics for further discussion to address caregivers’ potential unmet needs: psychological distress, health literacy, physical health, family support, financial burden, and practical difficulties (e.g. legal affairs). Caregivers could raise additional topics if required. In the attention control group, caregivers received three outcalls at the same time points as those in the intervention group (mean call duration: 3 min, 22 min; respectively). These outcalls were conducted by trained research personnel who supplied caregivers with the 13 11 20 number to self-initiate contact if needed, no other information or support was provided.

### Measures

Demographic characteristics were collected at baseline and included information on the caregiver (age, gender, postcode, type of relationship to the patient, living situation, household size, level of education) and patient (age, gender, postcode, treatment type, cancer diagnosis). Details of the measures used for caregiver and patient outcomes have been published [18]. Briefly, the primary outcome (caregiver burden) was measured using the Zarit Burden Interview (ZBI), which consists of 22 items [20]. A total score is calculated through summing individual item scores (range from 0 to 88) with higher scores indicating greater burden. Scores of 24 and above have been found to be indicative of risk for depression [21]. Secondary outcome measures for caregivers and patients were the Centre of Epidemiologic Studies – Depression scale (CES-D) [22] to measure depressive symptoms in caregivers and patients. This instrument consists of 20 items, each rated on a 4-point scale ranging from ‘rarely or none of the time’ to ‘most or all of the time’. The Supportive Care Needs Survey for Partners & Caregivers (SCNS-P&C, 45 items) [23], and the Supportive Care Needs Survey (SCNS-SF34, 34 items) [24] were used to assess the perceived needs of caregivers and cancer patients respectively. Items on both tools are rated on a 5-point scale ranging from ‘not applicable’ to ‘high need’. The Health Literacy Questionnaire (HLQ) [25] was used to assess caregivers’ and patients’ ability to obtain, understand and use health information. The HLQ consists of 44 items, each rated on a 5-point scale ranging from ‘cannot do’ to ‘very easy’. The health education impact Questionnaire (heiQ) [26] was used to measure self-empowerment. The heiQ contains 40 items, each rated on a 4-point scale ranging from ‘strongly disagree’ to ‘strongly agree’. The self-esteem subscale of the Caregiver Reaction Assessment (CRA, 7 items) was used to measure positive aspects of caregiving on a 5-point scale ranging from ‘strongly disagree’ to ‘strongly agree’ [27]. A self-designed utility assessment was included in the 1 month post-intervention survey to evaluate caregivers’ perceptions of the outcall program (i.e. ‘I feel it was worth my time and effort to take part in the outcall program’).

### Sample size calculation

The primary outcome was change in caregiver burden as measured by the ZBI. A sample size of 180 dyads (90 per group) at the end of the trial period was estimated to detect a moderate effect size (*d* = 0.5), which corresponds to a difference between the treatment groups of 6.3 units on the ZBI Total score, with 90% power and alpha = 0.05 (two-sided).

### Data analysis

An intention to treat analysis was performed and analyses were undertaken using GenStat and Stata. A mixed model analysis, using the restricted maximum likelihood method (REML), was used to calculate the between and within caregiver components of variance and the predicted main-effect means for study group, time (baseline and one-month post intervention) and the two-way interaction predicted means. A difference between the research groups in the change in caregiver burden was claimed if the F-test for the two-way interaction was significant (*P* < 0.05). Mixed model analyses were also used to analyse all secondary caregiver endpoints (CRA, CES-D, heiQ domains, HLQ subscales). Data from the 45-item SCNS-P&C were summarised as the total number of (moderate/high) unmet needs [7, 23] and a variance-stabilising square-root transformation was applied in mixed model analyses of this endpoint.

## Results

### Caregiver and patient sample

**Fig. 1:**
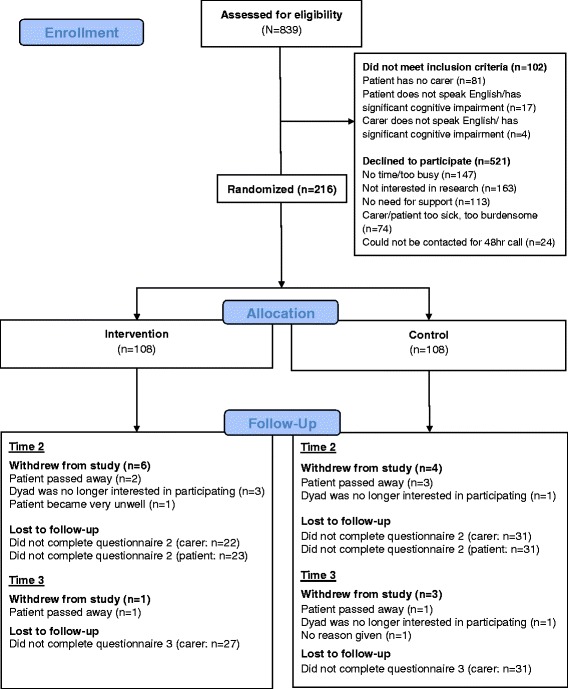
Consort diagram showing recruitment of patient/caregiver dyads into the PROTECT study

Overall, 839 patient/caregiver dyads were approached for study participation, of which 737 were eligible to participate and 216 (29%) provided informed consent and were included in each analysis. Dyads were randomized into intervention (*n* = 108) and attention control (*n* = 108) groups (Fig. 1).

The overall attrition rate at 1-month post intervention was 30% (intervention, 27%; control, 32%). Participants’ demographic characteristics are provided in Table 1. At baseline, there were no significant differences between groups in participants’ demographic or clinical characteristics.

**Table 1:** Baseline demographic and clinical characteristics of patients and caregivers in the PROTECT trial by study group (*N* = 216)

Characteristics	Control	Intervention	*P*
Patient demographics			
Age, mean (SD), years	59.8 (12.4)	58.8 (12.1)	.58
Gender, n (%)			.58
Male	49 (45.4)	45 (41.7)	
Female	59 (54.6)	63 (58.3)	
Clinical data			
Type of Cancer, n (%)			.21
Solid	92 (85.2)	98 (90.7)	
Haematological	16 (14.8)	10 (9.3)	
Treatment regimen, n (%)			.85
Chemotherapy	40 (37.0)	42 (38.9)	
Radiotherapy	38 (35.2)	34 (31.5)	
Chemo/Radio combined	30 (27.8)	32 (29.6)	
Caregiver demographics			
Age, mean (SD), years	56.3 (14.2)	57.2 (11.6)	.59
Gender, n (%)			.27
Male	42 (38.9)	50 (46.3)	
Female	66 (61.1)	58 (53.7)	
Indigeneity- Aboriginal/Torres Strait Islander, n (%)			–
Yes	0 (0)	0 (0)	
No	104 (100.0)	92 (100.0)	
Country of birth, n (%)			.46
Australia	84 (80.8)	83 (84.7)	
Other	20 (19.2)	15 (15.3)	
Lives with patient, n (%)			.85
Yes	90 (83.3)	91 (84.3)	
No	18 (16.7)	17 (15.7)	
Relationship to patient, n (%)			.87
Spouse/partner	85 (78.7)	86 (79.6)	
Other (e.g. parent, adult child, friend)	23 (21.3)	22 (20.4)	
Household size, mean (SD)			
Number of people ≥18 years	2.2 (0.7)	2.2 (0.7)	.85
Number of people <18 years	0.4 (0.9)	0.4 (0.9)	.88
Total household size	2.6 (1.1)	2.6 (1.2)	.81
Education status, n (%)			.85
Primary/secondary school	48 (44.9)	47 (43.5)	
Certificate/Diploma	27 (25.2)	25 (23.1)	
University Degree	32 (29.9)	36 (33.3)	

### Caregiver outcomes

Analyses of outcome variables for caregivers are summarized in Table 2.

**Table 2:** -Time (BL = Baseline, M1 = Month 1, M6 = Month 6) by treatment means for outcome variables for caregiver

Outcome	Control			Intervention			∆ = Intervention - Control		
	Mean	SE^a^	n (or *P*-value)^b^	Mean	SE	n (or *P*-value)	∆	SED^c^	*P*_value
ZBI^e^									
BL	18.05	1.17	108	18.99	1.17	107	0.95	1.65	0.568
M1	17.81	1.31	73	19.39	1.27	82	1.58	1.82	0.386
M6	17.20	1.33	69	18.46	1.31	73	1.26	1.87	0.500
M1-BL	−0.24	1.13	0.835	0.40	1.09	0.713			
M6-BL	−0.85	1.16	0.464	−0.53	1.14	0.639			
M6-M1	−0.61	1.23	0.620	−0.93	1.16	0.422			0.921^d^
CRA^f^ Total									
BL	30.07	0.44	108	30.80	0.44	105	0.73	0.62	0.243
M1	29.80	0.50	73	29.58	0.49	80	−0.22	0.70	0.750
M6	29.76	0.52	66	29.66	0.51	70	−0.09	0.73	0.897
M1-BL	−0.27	0.49	0.578	−1.22	0.48	0.012			
M6-BL	−0.31	0.51	0.538	−1.13	0.50	0.025			
M6-M1	−0.04	0.54	0.940	0.09	0.52	0.867			0.320
SCNS-P&C^g^ (√ transformed)									
BL	1.14	0.13	108	1.63	0.13	108	0.49	0.18	0.007
M1	1.01	0.15	75	0.92	0.14	83	−0.09	0.20	0.659
M6	0.41	0.15	68	0.53	0.15	73	0.12	0.21	0.577
M1-BL	−0.13	0.15	0.401	−0.71	0.15	<0.001			
M6-BL	−0.73	0.16	<0.001	−1.10	0.16	<0.001			
M6-M1	−0.60	0.17	0.001	−0.39	0.16	<0.001			0.023
CES-D^h^ Total									
BL	11.46	0.91	107	13.24	0.91	108	1.79	1.29	0.168
M1	10.98	1.02	75	12.32	0.99	82	1.34	1.42	0.347
M6	10.62	1.04	68	12.04	1.02	73	1.42	1.46	0.334
M1-BL	−0.48	0.89	0.592	−0.93	0.85	0.280			
M6-BL	−0.83	0.92	0.369	−1.20	0.89	0.180			
M6-M1	−0.36	0.97	0.715	−0.27	0.92	0.765			0.926
heiQ^i^ Dom1 HDB									
BL	2.90	0.07	107	2.83	0.07	108	−0.06	0.10	0.548
M1	3.05	0.08	75	2.81	0.08	82	−0.24	0.11	0.034
M6	2.96	0.08	68	2.92	0.08	73	−0.04	0.12	0.749
M1-BL	0.15	0.07	0.026	−0.03	0.07	0.680			
M6-BL	0.06	0.07	0.379	0.09	0.07	0.202			
M6-M1	−0.09	0.07	0.224	0.11	0.07	0.105			0.080
heiQ Dom2 PAEL									
BL	3.15	0.05	108	3.05	0.05	108	−0.10	0.07	0.157
M1	3.18	0.06	75	3.02	0.05	82	−0.17	0.08	0.035
M6	3.22	0.06	68	3.06	0.06	73	−0.16	0.08	0.053
M1-BL	0.03	0.05	0.464	−0.03	0.05	0.509			
M6-BL	0.07	0.05	0.166	0.01	0.05	0.782			
M6-M1	0.03	0.05	0.518	0.04	0.05	0.377			0.564
heiQ Dom3 ED									
BL	1.70	0.06	106	1.78	0.06	106	0.09	0.08	0.303
M1	1.73	0.07	75	1.95	0.06	82	0.22	0.09	0.015
M6	1.68	0.07	67	1.91	0.07	73	0.23	0.10	0.016
M1-BL	0.03	0.06	0.653	0.17	0.06	0.005			
M6-BL	−0.02	0.06	0.766	0.13	0.06	0.036			
M6-M1	−0.05	0.07	0.488	−0.04	0.06	0.537			0.144
heiQ Dom4 SMI									
BL	3.17	0.04	106	3.16	0.04	108	−0.01	0.06	0.859
M1	3.13	0.05	75	3.07	0.04	82	−0.06	0.06	0.354
M6	3.22	0.05	68	3.14	0.05	73	−0.08	0.07	0.209
M1-BL	−0.04	0.04	0.418	−0.08	0.04	0.047			
M6-BL	0.06	0.05	0.199	−0.01	0.04	0.749			
M6-M1	0.09	0.05	0.051	0.07	0.05	0.124			0.489
heiQ Dom5 CAA									
BL	3.38	0.05	105	3.31	0.05	104	−0.07	0.06	0.268
M1	3.30	0.05	75	3.20	0.05	82	−0.11	0.07	0.124
M6	3.38	0.05	67	3.25	0.05	72	−0.13	0.07	0.083
M1-BL	−0.08	0.05	0.117	−0.12	0.05	0.016			
M6-BL	0.00	0.05	0.980	−0.06	0.05	0.256			
M6-M1	0.08	0.05	0.145	0.06	0.05	0.248			0.701
heiQ Dom6 STA									
BL	3.17	0.04	102	3.15	0.04	103	−0.02	0.06	0.794
M1	3.04	0.05	74	3.01	0.05	83	−0.04	0.07	0.560
M6	3.17	0.05	66	3.09	0.05	73	−0.08	0.07	0.262
M1-BL	−0.12	0.05	0.015	−0.15	0.05	0.002			
M6-BL	0.00	0.05	0.997	−0.06	0.05	0.202			
M6-M1	0.12	0.05	0.026	0.08	0.05	0.108			0.672
heiQ Dom7 SIS									
BL	3.23	0.05	106	3.12	0.05	104	−0.11	0.07	0.114
M1	3.08	0.05	75	2.98	0.05	83	−0.10	0.08	0.212
M6	3.24	0.06	68	3.05	0.06	73	−0.19	0.08	0.016
M1-BL	−0.15	0.05	0.003	−0.13	0.05	0.006			
M6-BL	0.01	0.05	0.778	−0.07	0.05	0.191			
M6-M1	0.16	0.05	0.003	0.07	0.05	0.193			0.392
heiQ Dom8 HSN									
BL	3.27	0.05	105	3.17	0.05	103	−0.10	0.07	0.153
M1	3.23	0.05	75	3.07	0.05	83	−0.16	0.08	0.034
M6	3.39	0.06	67	3.15	0.05	73	−0.24	0.08	0.003
M1-BL	−0.04	0.05	0.409	−0.10	0.05	0.032			
M6-BL	0.12	0.05	0.015	−0.02	0.05	0.753			
M6-M1	0.16	0.05	0.002	0.09	0.05	0.086			0.144
HLQ^j^ S1									
BL	3.24	0.05	105	3.11	0.05	103	−0.13	0.07	0.083
M1	3.25	0.06	73	3.15	0.06	83	−0.10	0.08	0.208
M6	3.30	0.06	67	3.17	0.06	73	−0.13	0.08	0.109
M1-BL	0.01	0.05	0.913	0.03	0.05	0.492			
M6-BL	0.06	0.05	0.220	0.06	0.05	0.239			
M6-M1	0.06	0.05	0.288	0.03	0.05	0.604			0.892
HLQ S2									
BL	3.15	0.04	106	3.10	0.04	105	−0.05	0.06	0.388
M1	3.15	0.05	74	3.09	0.05	83	−0.06	0.07	0.392
M6	3.23	0.05	67	3.19	0.05	73	−0.04	0.07	0.552
M1-BL	−0.01	0.05	0.880	−0.01	0.04	0.785			
M6-BL	0.08	0.05	0.107	0.09	0.05	0.058			
M6-M1	0.08	0.05	0.097	0.10	0.05	0.034			0.970
HLQ S3									
BL	2.96	0.05	107	2.89	0.05	105	−0.07	0.07	0.282
M1	2.95	0.05	74	2.91	0.05	83	−0.04	0.07	0.616
M6	2.99	0.05	67	2.91	0.05	72	−0.08	0.08	0.286
M1-BL	−0.02	0.05	0.758	0.02	0.05	0.692			
M6-BL	0.03	0.05	0.558	0.02	0.05	0.695			
M6-M1	0.05	0.05	0.405	0.00	0.05	0.987			0.815
HLQ S4									
BL	3.19	0.05	106	3.04	0.05	105	−0.16	0.07	0.019
M1	3.12	0.05	74	3.02	0.05	83	−0.10	0.07	0.166
M6	3.23	0.05	68	3.04	0.05	73	−0.19	0.07	0.010
M1-BL	−0.07	0.05	0.119	−0.02	0.04	0.721			
M6-BL	0.04	0.05	0.382	0.00	0.05	0.940			
M6-M1	0.11	0.05	0.024	0.02	0.05	0.682			0.381
HLQ S5									
BL	2.90	0.05	104	2.86	0.05	104	−0.04	0.07	0.587
M1	2.89	0.06	73	2.88	0.05	83	−0.01	0.08	0.878
M6	2.95	0.06	67	2.91	0.06	73	−0.04	0.08	0.587
M1-BL	−0.01	0.05	0.802	0.01	0.05	0.788			
M6-BL	0.05	0.05	0.372	0.04	0.05	0.412			
M6-M1	0.06	0.06	0.282	0.03	0.05	0.578			0.903
HLQ S6									
BL	4.22	0.06	106	4.23	0.06	104	0.01	0.09	0.863
M1	4.19	0.07	74	4.28	0.07	83	0.10	0.10	0.322
M6	4.28	0.07	68	4.18	0.07	73	−0.10	0.10	0.331
M1-BL	−0.03	0.07	0.626	0.05	0.07	0.470			
M6-BL	0.06	0.07	0.388	−0.05	0.07	0.454			
M6-M1	0.09	0.07	0.207	−0.10	0.07	0.158			0.168
HLQ S7									
BL	4.14	0.06	104	4.12	0.06	104	−0.03	0.09	0.772
M1	4.10	0.07	73	4.21	0.07	83	0.11	0.10	0.259
M6	4.19	0.07	68	4.14	0.07	73	−0.05	0.10	0.629
M1-BL	−0.04	0.06	0.498	0.09	0.06	0.143			
M6-BL	0.05	0.07	0.488	0.02	0.06	0.717			
M6-M1	0.09	0.07	0.200	−0.07	0.06	0.306			0.192
HLQ S8									
BL	4.15	0.06	105	4.13	0.06	104	−0.03	0.09	0.774
M1	4.08	0.07	73	4.18	0.07	83	0.09	0.10	0.341
M6	4.19	0.07	68	4.13	0.07	73	−0.05	0.10	0.614
M1-BL	−0.07	0.07	0.333	0.05	0.07	0.473			
M6-BL	0.03	0.07	0.657	0.01	0.07	0.928			
M6-M1	0.10	0.08	0.192	−0.04	0.07	0.559			0.336
HLQ S9									
BL	4.31	0.05	104	4.30	0.05	104	−0.01	0.08	0.916
M1	4.27	0.06	74	4.37	0.06	83	0.10	0.08	0.234
M6	4.32	0.06	68	4.37	0.06	73	0.05	0.09	0.563
M1-BL	−0.04	0.06	0.512	0.07	0.06	0.197			
M6-BL	0.00	0.06	0.948	0.06	0.06	0.279			
M6-M1	0.04	0.06	0.504	−0.01	0.06	0.884			0.390

^a^ *SE* Standard Error of the Mean or a difference in Time Means

^b^ Sample sizes (n) for means and *p*-values for time differences within treatment groups

^c^ *SED* Standard Error of the Difference in Treatment group means within a Time

^d^ P-value for the 2 degree-of-freedom test for a Time by Treatment group interaction

^e^ *ZBI* Zarit Burden Interview

^f^ *CRA* Caregiver Reaction Assessment

^g^ *SCNS – P&C* Supportive Care Needs Survey – Partner & Caregivers

^h^ *CES-D* Centre for Epidemiologic Studies – Depression

^i^ *heiQ* health education impact Questionnaire: Domain 1 – health directed behaviour (HDB), Domain 2 – positive and active engagement in life (PAEL), Domain 3 – emotional distress (ED), Domain 4 – self monitoring and insight (SMI), Domain 5 – constructive attitudes and approaches (CAA), Domain 6 – skills and technique acquisition (STA), Domain 7 – social integration and support (SIS), Domain 8 – health service navigation (HSN)

^j^ *HLQ* Health Literacy Questionnaire: S1 – feeling understood and supported by healthcare providers, S2 – having sufficient information to manage my health, S3 – actively managing my health, S4 – social support for health, S5 – appraisal of health information, S6 – ability to actively engage with health care providers, S7 – navigating the health care system, S8 – ability to find good health information, S9 – understanding health information well enough to know what to do

#### Caregiver burden

At baseline, the primary outcome variable caregiver burden (ZBI Total Score), in both the intervention group (*M* = 18.99, SE = 1.17) and the control group (*M* = 18.05, SE = 1.17) was low. The intervention had no significant effect on caregiver burden (*P* = 0.921). The estimated between-individual and within-individual variance components were 97.2 and 50.4 respectively (ICC = 0.659).

#### Caregiver unmet needs

Unmet needs declined in both groups over the course of the intervention period (*P* < 0.001) and there was a significant interaction between study group and time (*P* = 0.023) with a greater decline from baseline to month 1 in the intervention group compared to the control group (*t* = −2.703; df = 312.3; *P* = 0.007). The decline from baseline to month 6 appeared to be greater in the intervention group but this was not significant at the conventional 5% level (*t* = −1.661; df = 312.3; *P* = 0.098). Back-transformed means (95% confidence intervals) at baseline, month 1 and month 6 were 1.30 (0.80, 1.94), 1.02 (0.52, 1.69) and 0.17 (0.01, 0.51) respectively in the control group, and 2.66 (1.91, 3.54), 0.85 (0.42, 1.44) and 0.28 (0.06, 0.68) respectively in the intervention group.

#### Caregiver self-empowerment (heiQ) and health literacy

There were no statistically significant differences between the study groups in their changes over time in heiQ subscales as indicated by the outcomes of F-tests for time by treatment interactions (Table 2). No significant effects were found for health literacy subscales (data not shown).

#### Caregiver self-esteem

Caregiver self-esteem (CRA) declined in both groups from baseline to months 1 and 6 (*P* = 0.045) and there was no significant difference between the groups in their declines over time (*P* = 0.320).

#### Depressive symptoms and caregivers at risk for depression

No significant effects were found for depressive symptoms (CES-D Total Score). A subsample of caregivers had burden scores of 24 or greater (intervention, *n* = 37; control, *n* = 31), which indicated risk for depression. Caregivers in these groups did not differ with respect to baseline demographic and clinical characteristics (Table 3). Significant demographic differences were found when comparing caregivers at risk for depression (ZBI ≥ 24) with those not at risk (ZBI < 24). Caregivers at risk were about 6 years younger (52.9 vs 58.4 years; *P =* 0.003) and had more people in their households (2.9 vs 2.5 mean household size; *P =* 0.04) than those not at risk (Table 4). For caregivers at risk, the intervention had a significant effect on *having sufficient information to manage caregivers health* (*P* = 0.040). Post hoc analyses showed an increase in caregivers’ confidence that they had sufficient information to manage their health (HLQ Scale 2) between baseline and 6 months (*P* = 0.002) and between 1 month and 6 months (*P* = 0.009) in the intervention group but no such changes were observed in the control group (*P* > 0.30). No significant differences between the groups, in their changes over time, were observed for the other caregiver outcome variables.

**Table 3:** –Baseline demographic and clinical characteristics of patients and caregivers in the PROTECT trial by study group: Dyads including caregivers at risk of depression (*n* = 68)

Characteristics	Control	Intervention	*P*
Patient demographics			
Age, mean (SD), years	59.1 (12.9)	57.7 (11.6)	.63
Gender, n (%)			.81
Male	15 (48.4)	19 (51.4)	
Female	16 (51.6)	18 (48.6)	
Clinical data			
Type of Cancer, n (%)			.09
Solid	24 (77.4)	34 (91.9)	
Haematological	7 (22.6)	3 (8.1)	
Treatment regimen, n (%)			.17
Chemotherapy	14 (45.2)	13 (35.1)	
Radiotherapy	11 (35.5)	9 (24.3)	
Chemo/Radio combined	6 (19.4)	15 (40.5)	
Caregiver demographics			
Age, mean (SD), years	53.6 (12.9)	52.3 (12.3)	.67
Gender, n (%)			.67
Male	11 (35.5)	15 (40.5)	
Female	20 (64.5)	22 (59.5)	
Indigeneity- Aboriginal/Torres Strait Islander, n (%)			–
Yes	0 (0)	0 (0)	
No	31 (100.0)	37 (100.0)	
Country of birth, n (%)			.21
Australia	23 (79.3)	29 (90.6)	
Other	6 (20.7)	3 (9.4)	
Lives with patient, n (%)			.52
Yes	26 (83.9)	33 (89.2)	
No	5 (16.1)	4 (10.8)	
Relationship to patient, n (%)			.33
Spouse/partner	23 (74.2)	31 (83.8)	
Other (e.g. parent, adult child, friend)	8 (25.8)	6 (16.2)	
Household size, mean (SD)			
Number of people ≥18 years	2.3 (0.8)	2.2 (0.6)	.65
Number of people <18 years	0.6 (1.0)	0.6 (1.2)	.97
Total household size	2.9 (1.3)	2.8 (1.3)	.84
Education status, n (%)			.99
Primary/secondary school	11 (36.7)	13 (35.1)	
Certificate/Diploma	10 (33.3)	13 (35.1)	
University Degree	9 (30.0)	11 (39.7)

**Table 4:** Baseline demographic and clinical characteristics of patients and caregivers in the PROTECT trial: Comparison of dyads including caregivers at risk of depression with those not at risk (*n* = 215^a^)

Characteristics	Caregivers not at risk (*n* = 147)	Caregivers at risk (*n* = 68)	*P*
Patient demographics			
Age, mean (SD), years	59.6 (12.3)	58.3 (12.1)	.47
Gender, n (%)			.18
Male	59 (40.1)	34 (50.0)	
Female	88 (59.9)	34 (50.0)	
Clinical data			
Type of Cancer, n (%)			.42
Solid	131 (89.1)	58 (85.3)	
Haematological	16 (10.9)	10 (14.7)	
Treatment regimen, n (%)			.74
Chemotherapy	55 (37.4)	27 (39.7)	
Radiotherapy	51 (34.7)	20 (29.4)	
Chemo/Radio combined	41 (27.9)	21 (30.9)	
Caregiver demographics			
Age, mean (SD), years	58.4 (12.8)	52.9 (12.5)	.003
Gender, n (%)			.36
Male	66 (44.9)	26 (38.2)	
Female	81 (55.1)	42 (61.8)	
Indigeneity- Aboriginal/Torres Strait Islander, n (%)			–
Yes	0 (0)	0 (0)	
No	147 (100.0)	68 (100.0)	
Country of birth, n (%)			.51
Australia	114 (81.4)	52 (85.2)	
Other	26 (18.6)	9 (14.8)	
Lives with patient, n (%)			.41
Yes	121 (82.3)	59 (86.8)	
No	26 (17.7)	9 (13.2)	
Relationship to patient, n (%)			.93
Spouse/partner	116 (78.9)	54 (79.4)	
Other (i.e. parent, adult child, friend)	31 (21.1)	14 (20.6)	
Household size, mean (SD)			
Number of people ≥18 years	2.2 (0.8)	2.3 (0.7)	.35
Number of people <18 years	0.4 (0.7)	0.6 (1.1)	.10
Total household size	2.5 (1.0)	2.9 (1.3)	.04
Education status, n (%)			.06
Primary/secondary school	70 (47.6)	24 (35.8)	
Certificate/Diploma	29 (19.7)	23 (34.3)	
University Degree	48 (32.7)	20 (29.9)	

^a^ One caregiver in the intervention condition had missing data for the ZBI and, therefore, wasn’t included in the analysis

For patients associated with caregivers at risk of depression, there was a significant decline in emotional distress from baseline (BL) to 1 month (M1) (*P* = 0.001). Investigation of the significant three-way interaction (*P* = .027) indicated a significant decrease in emotional distress in the control group (BL mean = 2.592, M1 mean = 2.046, SED = 0.133, *P* < .001) but a non-significant decrease in the intervention group (BL mean = 2.487, M1 mean = 2.288, SED = 0.117, *P* = .089). No other statistically significant effects were found.

### Patient outcomes

No significant differences between the groups, in their changes over time were observed for patients’ depressive symptoms, unmet needs or health literacy (Additional file 1: Table s1).

A significant improvement in positive and active engagement in life (heiQ, domain 2) among patients in the control group, but not the intervention group, was noted (*t* = 2.972; df = 168.4; *P* = 0.003). No significant effects were found for other subscales of the heiQ.

### Self-initiated contact to the 13 11 20 service by caregivers in the attention control group

Of the 108 participants in the attention control group, seven caregivers (6%) initiated contact to the 13 11 20 service between outcalls. Of those, two were repeat callers living in outer regional areas.

### Perceptions of caregivers on the outcall program

**Fig. 2:**
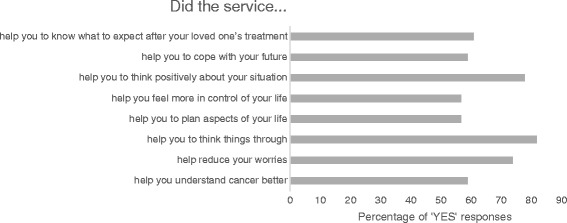
Caregivers’ perception of the value of the telephone outcall program

Caregivers in the intervention group were asked in what way the outcall program had helped them in their role as a caregiver (Fig. 2). Most caregivers reported that the service had helped them to reduce their worries (74%), to think positively about their situation (78%), and to think things through (82%).

## Discussion

This study tested the impact of a telephone outcall intervention delivered by two Australian Cancer Council 13 11 20 services on the psychological distress of caregivers of newly diagnosed cancer patients. Results indicated that the outcall program did not reduce caregivers’ overall caregiver burden. This may be due to the inclusion of caregivers with various levels of burden in this study and the fact that burden levels at baseline, for the majority of caregivers, were reasonably low, limiting the detection of changes over time.

While the outcall program had no significant impact on depressive symptoms in caregivers, it was associated with an increase in health literacy in a subgroup of caregivers who were at increased risk for depression at baseline (ZBI scores of ≥24). For these caregivers, the intervention significantly increased caregivers’ confidence in having sufficient information to manage their own (as opposed to the patient’s) health. It is possible that these caregivers had low health literacy, hence the high burden levels, and the informational component of the intervention was able to meet caregivers’ needs in this domain. We also found that caregivers ‘at risk’ were those who were younger in age and had more people residing in their households than those not at risk. Since this subgroup was relatively small (intervention, *n* = 37; control, *n* = 31) our ability to detect a significant impact, if in fact it exists, of the outcall program was impaired. Future research may consider screening caregivers for age, household size, and associated burden such as the care of young children or employment, as those may benefit from interventions of this type.

The outcall program produced positive changes over time with a greater reduction in the number of caregivers’ reported unmet needs. The six topics raised for discussion by the 13 11 20 nurses at each outcall were specifically chosen to address caregiver’s supportive care needs previously reported in the literature [4]. Researchers have stressed the importance of targeting caregivers’ needs to minimise long-term health problems [5, 8, 9]. Therefore, our program, which addressed caregivers’ unique needs at the early stages of the cancer trajectory, may be a good approach in preventing long-term negative health outcomes in caregivers. This hypothesis warrants further investigation.

We hypothesised that improved caregiver outcomes would impact positively on patient health and well-being. However, the outcall program had no significant effect on patient outcomes and our subgroup of burdened caregivers (ZBI >24) was possibly underpowered to detect significant improvements in patient outcomes. Targeting dyad interventions to specific subgroups (e.g. significantly burdened caregivers) may be more effective in improving patient wellbeing, however more research is needed to support this argument.

The outcall program was reported as beneficial by trial participants. The majority of caregivers stated that the outcalls helped them to reduce their worries and think more positively about their situation. These findings suggest that the program was acceptable to caregivers and may represent a feasible approach to provide information and support to a targeted population.

Limitations of this study included a modest recruitment rate of 29%, which reflects the difficulty of enrolling cancer dyads into randomised trials consistently reported in the literature [28–31]. It is possible that eligible dyads who experienced significant burden declined participation due to the perceived burden of taking part in research, even though the intervention may have been of benefit to them. The overall attrition rate (30%) in this study at month 1 was higher than the estimated attrition rate of 20%. Further, we cannot rule out that caregivers in the attention control group actively sought support elsewhere as their unmet needs also declined from baseline to month 1. Despite these limitations, the findings suggest that access to support services for caregivers, who are at risk for depression, can ameliorate the demands of caregiving and potentially improve quality of life outcomes; telephone information support services are a feasible approach to providing such access and warrant further investigation in this high risk group.

## Conclusion

While the 4-month telephone outcall program did not reduce caregiver burden, it was effective in reducing caregivers’ reported unmet supportive care needs. One third of caregivers were found to be at risk of depression and this was particularity the case in younger caregivers and those residing in larger households. Findings suggest that the provision of cancer information and support via a telephone service may represent a feasible approach to address caregivers’ unique needs at the early stages of the cancer trajectory. However, the role of a telephone service to help prevent medium- and long-term negative health outcomes in this population group warrants further research.

## Additional file

Additional file 1: Table S1. Patient outcomes. (DOCX 14 kb)

## Abbreviations

ANZCTR: Australian New Zealand Clinical Trials Registry
BL: Baseline
CES-D: Centre of Epidemiologic Studies – Depression scale
CI: Confidence Interval
CRA: Caregiver Reaction Assessment
df: Degree of freedom
heiQ: Health education impact Questionnaire
HLQ: Health Literacy Questionnaire
M: Mean
M1: 1 Month
M6: 6 Month
RCT: Randomized Controlled Trial
REML: Restricted Maximum Likelihood Method
SCNS-P&C: Supportive Care Needs Survey for Partners & Caregivers
SCNS-SF34: Supportive Care Needs Survey
SD: Standard deviation
SE: Standard Error
SED: Standard Error of the Difference
ZBI: Zarit Burden Interview
