# Supplementary material for: Efficacy of a telephone outcall program to reduce caregiver burden among caregivers of cancer patients [PROTECT]: a randomised controlled trial
Source: BMC Cancer. 2018 Jan 8;18:59. doi: 10.1186/s12885-017-3961-6 (PMC5759190; doi:10.1186/s12885-017-3961-6)
Supplement: Additional file 1: Table S1. — Patient outcomes. (DOCX 14 kb) [file 12885_2017_3961_MOESM1_ESM.docx]

**Efficacy of a telephone outcall program to reduce caregiver burden among caregivers of cancer patients [PROTECT]: a randomised controlled trial**

**Table S1: Patient outcomes.**

| **F-test p-values for patient outcome variables** | **Study condition** | **Time** | **Study condition by Time** |
| --- | --- | --- | --- |
| **Centre of Epidemiologic Studies – Depression scale (CES-D)** | | |  |
| CESD Total | 0.772 | 0.017 | 0.346 |
| **health education impact Questionnaire (heiQ)^1^** |  |  |  |
| heiQ Dom1 HDB | 0.519 | 0.813 | 0.172 |
| heiQ Dom2 PAEL | 0.781 | 0.132 | 0.003 |
| heiQ Dom3 ED | 0.274 | 0.022 | 0.961 |
| heiQ Dom4 SMI | 0.507 | 0.803 | 0.113 |
| heiQ Dom5 CAA | 0.797 | 0.583 | 0.760 |
| heiQ Dom6 STA | 0.373 | 0.357 | 0.369 |
| heiQ Dom7 SIS | 0.461 | 0.022 | 0.824 |
| heiQ Dom8 HSN | 0.658 | 0.085 | 0.127 |
| **The Health Literacy Questionnaire (HLQ)^2^** |  |  |  |
| HLQ S1 | 0.600 | 0.857 | 0.204 |
| HLQ S2 | 0.324 | 0.849 | 0.824 |
| HLQ S3 | 0.379 | 0.348 | 0.343 |
| HLQ S4 | 0.376 | 0.048 | 0.589 |
| HLQ S5 | 0.856 | 0.338 | 0.443 |
| HLQ S6 | 0.632 | 0.242 | 0.172 |
| HLQ S7 | 0.563 | 0.423 | 0.978 |
| HLQ S8 | 0.840 | 0.422 | 0.871 |
| HLQ S9 | 0.557 | 0.876 | 0.263 |
| **Supportive Care Needs Survey**  **(SCNS-SF34)^3^** | |  |  |
| SF34 P1 Psyc | 0.887 | <0.001 | 0.808 |
| SF34 P2 HSI | 0.037 | <0.001 | 0.400 |
| SF34 P3 PDL | 0.553 | <0.001 | 0.467 |
| SF34 P4 PCS | 0.301 | <0.001 | 0.283 |
| SF34 P5 SX | 0.498 | 0.844 | 0.172 |

^1^heiQ = health education impact Questionnaire: Domain 1 – health directed behaviour (HDB), Domain 2 – positive

and active engagement in life (PAEL), Domain 3 – emotional distress (ED), Domain 4 – self monitoring and insight

(SMI), Domain 5 – constructive attitudes and approaches (CAA), Domain 6 – skills and technique acquisition (STA),

Domain 7 – social integration and support (SIS), Domain 8 – health service navigation (HSN)

^2^HLQ = Health Literacy Questionnaire: S1 – feeling understood and supported by healthcare providers, S2 – having

sufficient information to manage my health, S3 – actively managing my health, S4 – social support for health,

S5 – appraisal of health information, S6 – ability to actively engage with health care providers, S7 – navigating the

health care system, S8 – ability to find good health information, S9 – understanding health information well enough

to know what to do

^3^SCNS-SF34 = Supportive Care Needs Survey-SF34: P1 – psychological needs (Psyc), P2 – health system and

information needs (HIS), P3 – physical & daily living needs (PDL), P4 – patient care & support needs (PCS),

P5 – sexuality needs (SX)
